# A hybrid model of tumor growth and angiogenesis: *In silico* experiments

**DOI:** 10.1371/journal.pone.0231137

**Published:** 2020-04-10

**Authors:** Caleb M. Phillips, Ernesto A. B. F. Lima, Ryan T. Woodall, Amy Brock, Thomas E. Yankeelov

**Affiliations:** 1 Oden Institute for Computational Engineering and Sciences, The University of Texas at Austin, Austin, TX, United States of America; 2 Department of Biomedical Engineering, The University of Texas at Austin, Austin, TX, United States of America; 3 Livestrong Cancer Institutes, The University of Texas at Austin, Austin, TX, United States of America; 4 Department of Diagnostic Medicine, The University of Texas at Austin, Austin, TX, United States of America; 5 Department of Oncology, The University of Texas at Austin, Austin, TX, United States of America; Yale University School of Medicine, UNITED STATES

## Abstract

Tumor associated angiogenesis is the development of new blood vessels in response to proteins secreted by tumor cells. These new blood vessels allow tumors to continue to grow beyond what the pre-existing vasculature could support. Here, we construct a mathematical model to simulate tumor angiogenesis by considering each endothelial cell as an agent, and allowing the vascular endothelial growth factor (VEGF) and nutrient fields to impact the dynamics and phenotypic transitions of each tumor and endothelial cell. The phenotypes of the endothelial cells (i.e., tip, stalk, and phalanx cells) are selected by the local VEGF field, and govern the migration and growth of vessel sprouts at the cellular level. Over time, these vessels grow and migrate to the tumor, forming anastomotic loops to supply nutrients, while interacting with the tumor through mechanical forces and the consumption of VEGF. The model is able to capture collapsing and breaking of vessels caused by tumor-endothelial cell interactions. This is accomplished through modeling the physical interaction between the vasculature and the tumor, resulting in vessel occlusion and tumor heterogeneity over time due to the stages of response in angiogenesis. Key parameters are identified through a sensitivity analysis based on the Sobol method, establishing which parameters should be the focus of subsequent experimental efforts. During the avascular phase (i.e., before angiogenesis is triggered), the nutrient consumption rate, followed by the rate of nutrient diffusion, yield the greatest influence on the number and distribution of tumor cells. Similarly, the consumption and diffusion of VEGF yield the greatest influence on the endothelial and tumor cell numbers during angiogenesis. In summary, we present a hybrid mathematical approach that characterizes vascular changes *via* an agent-based model, while treating nutrient and VEGF changes through a continuum model. The model describes the physical interaction between a tumor and the surrounding blood vessels, explicitly allowing the forces of the growing tumor to influence the nutrient delivery of the vasculature.

## Introduction

Tumor growth and development is dictated by the interaction of a myriad of events occurring at dramatically different spatial and temporal scales. At the intracellular scale, cell signaling results in gene and protein expression that promote cell events such as proliferation or migration. Cellular events are also governed by the availability of nutrients and interactions with specific proteins. Furthermore, the production and consumption of nutrients and proteins are based on the heterogeneity of the tumor and the surrounding vasculature at the tissue scale. Due to this complex, multiscale system, mathematical and computational models have been designed to describe the biological mechanisms that underlay tumor growth and treatment response. These models have aided in understanding the intricate interplay between phenomena at the cell [1–3], microenvironmental [4–6], and tissue scales [7–9]. Additionally, key features in tumor development such as tumor proliferation and apoptosis [10], nutrient availability [11], mechanical pressures [12, 13], and therapies [14–16] have been investigated and modeled, aspiring to marry experimental biology and mathematical methods to establish a data-informed, mathematical theory of tumor initiation and growth. The ultimate goal of these models is to uncover fundamental biology as well as provide predictions of tumor growth and treatment options that can be made specific for each individual patient [17, 18].

The dependence of events on different scales has motivated the development of mathematical models of tumor growth designed to capture the relationship between the subcellular, cellular, and tissue scales [19]. For example, Macklin *et al*. [20] developed a hybrid multiscale approach where the cellular dynamics and the macroscopic environment impact both the growth and development of the tumor. In particular, both nutrient availability at the macroscopic level and patient-specific measurements (based on histology data) at the subcellular scale govern the proliferation of tumor cells. The cellular scale is governed by an agent-based model (ABM) wherein cancer cells may divide, migrate, or die due to the local conditions of the environment, while the nutrient dynamics at the macroscopic scale are governed by a continuum (partial differential equation) model. By using a hybrid model, the macroscopic environment influences the decision-making process of individual cells, which are modeled as agents. Using this approach, the authors were able to match the tumor growth and calcification trends of the patient mammographic data, verifying both the ABM and its ability to recapitulate tumor heterogeneity. Rocha *et al*. [21] extended this multiscale approach to the subcellular scale, guiding tumor cell proliferation by both nutrient availability at the macroscopic scale and extracellular signal-regulated kinases at the subcellular scale. The main morphological features of solid tumors (e.g., the proliferative ring, the hypoxic region, and the necrotic core) are preserved in this avascular model. In Jiang *et al*. [8], reaction-diffusion equations govern the tissue scale and inform the protein expression of each cell, causing the cell to either proliferate, stay quiescent, or die. The model effectively predicts *in vitro* growth curves of tumor spheroids. Importantly, all of the above efforts characterized avascular tumor growth. Of course, once a tumor grows beyond a diameter of 0.2–1 mm [22, 23], the continued expansion of the tumor cannot be supported by only the diffusion of metabolites. Continued growth requires the delivery of oxygen and nutrients through new vasculature. Thus, for avascular models to remain informative past the initial stages of tumor development, they must be extended to incorporate the formation of new blood vessels, a process called angiogenesis.

Tumor angiogenesis is induced by growth factors released by hypoxic tumor cells, most notably the vascular endothelial growth factors (VEGF) [24, 25]. VEGF diffuses through the interstitial fluid and binds to the vascular endothelial growth factor receptors of pre-existing endothelial cells which then become activated, and migrate up the concentration gradient of VEGF, toward the tumor cells. These migratory cells, called tip cells [25, 26], guide the endothelial cells immediately adjacent them toward the tumor. The resulting, newly formed, blood vessels grow and mature and are characterized by branching, lumen formation, anastomosis formation, and establishment of blood flow [22, 23]. Once a vessel network is formed, the growth of the tumor can be accelerated by the newly available nutrients and may continue to proliferate and expand.

Computational models have been developed that incorporate angiogenesis using an agent-based approach. Sun et al. [27] developed a 2D multiscale ABM of brain cancer. At the subcellular scale, a system of ordinary differential equations (ODEs) govern the epidermal growth factor receptor pathway which, together with the availability of nutrients, govern the phenotypic transitions of the tumor cells at the cellular scale. At the tissue scale the growth of new blood vessels is based on a single endothelial tip cell, and the migration probabilities are based on the local VEGF and fibronectin concentrations. Olsen et al. [28] modeled tumor angiogenesis on a grid-based system, with new vessels sprouting and moving based on the local VEGF concentration. Endothelial cells are added to grid spaces at the end of the vessel to model growth and, after forming a closed loop, these vessels can deliver nutrients to the hypoxic tumor. At the cellular scale, each tumor cell is treated as an agent undergoing a decision-making process; for example, changing phenotypes based on the available nutrients, or migrating based on VEGF and available space. Other efforts in modeling angiogenesis include Cellular Potts models [29, 30], rule-based models of angiogenesis [31–35], agent-based approaches without tumor growth [36–38], and hybrid models that combine continuum and discrete approaches [31, 39–43].

We seek to build on these earlier efforts, that capture the effects of nutrient delivery from newly formed blood vessels, by including a physical mechanism guiding the interaction between the vasculature and the tumor. This physical mechanism allows for the modeling of vessel occlusion and collapse due to the proliferation of newly vascularized tumor regions. More specifically, the tumor angiogenic sprouting dynamics are modeled at the cellular scale by a discrete model, treating every cell as an individual agent that moves according to physical forces and chemical gradients, and allowing transitions between tip, stalk, and quiescent endothelial cells. Computing the physical forces between the tumor and endothelial cells provides a novel way to model vessel occlusion and collapse. The delivery and dispersion of nutrient and VEGF are modeled at the tissue scale by a continuum model, which is coupled with the ABM at the cell scale. The avascular component of the model is based on the work developed in [20]. To motivate the extension to this model, we begin with a brief biological background covering the main features of angiogenesis. This includes a discussion of VEGF secretion, tip cell selection, sprout and lumen formation, and branching of the newly formed blood vessels. We follow the biological background with the development of the ABM and the reaction-diffusion equations characterizing the cellular and tissue scales, respectively, as well as how they are coupled. Finally, results of several simulations are presented, and the major conclusions of the study are summarized.

## Biological background

Angiogenesis is the process by which new blood vessels sprout and develop from pre-existing blood vessels. Physiological angiogenesis is a tightly regulated process that is a critical part of maintaining nutrient delivery by creating new vessels during embryogenesis [44], wound healing [45], and bypassing blocked vessels [46]. Tumor-associated angiogenesis was first systematically investigated by Ide [47], and is now widely acknowledged as one of the fundamental hallmarks of cancer [48, 49]. The angiogenic response of endothelial cells is induced by VEGF and begins by the selection of a tip cell which guides the nascent vessel toward the tumor cells. Tip cells are the tip of the growing vessel and use long filopedia to navigate the surrounding environment. Behind tip cells, stalk cells proliferate to allow the developing vessel to lengthen and are essential in the formation of the lumen. Following the stalk cells, phalanx [50] cells provide rigidity and stability to the growing vessel. This tip-stalk-phalanx concept will be used to simulate vessel initiation, growth, and development. For more on these topics, the interested reader is referred to [26].

### VEGF released by hypoxic tumor cells

During the initial stages of tumor development, cancer cells proliferate rapidly and consume nutrients at an unsustainable rate [49]. Once the supply of glucose and oxygen is depleted, the cancer cells begin to become hypoxic and require a new supply of nutrients to continue proliferating. Hypoxic tumor cells then release VEGF into the surrounding areas which binds to the VEGF receptors on the cell membrane of endothelial cells lining the blood vessel walls [51]. This process induces an intracellular signaling event that initiates proliferation and migration of endothelial cells up the VEGF concentration gradient and ultimately leads to the development of new blood vessels. Endothelial cells that make up the pre-existing vasculature proximal to the tumor become activated due to the cell response to VEGF; however, not all activation of the epithelium leads to an endothelial cell expressing the tip cell phenotype [52]. Activation refers to the cell response to VEGF binding to the receptors causing the cell to express tip cell characteristics (filopedia, migratory, etc.).

### Lumen formation

As the stalk cells continue to proliferate and the vascular sprout [53, 54] elongates, a lumen forms, allowing blood flow through the vessel [55]. The most common mechanisms behind this are believed to be intercellular methods such as vacuolation and extracellular methods such as cell-cell repulsion [52, 56–59]. Vacuolation suggests that the lumen forms through intracellular vacuole coalescence or intercellular vacuole exocytosis. In cell-cell repulsion, during sprout growth the endothelial cells on opposite sides of the vessels begin to adhere to one another along the apical basal membrane, the side of the membrane of the cell facing the lumen [52, 60]. As the vascular sprout continues to grow, proteins polarize the apical membrane of the adhered endothelial cells, causing a repulsion between them [60]. This repulsion allows blood to flow through the newly-formed lumen, enabling the delivery of (for example) glucose and oxygen to the tumor [48, 49]. More detailed reviews of vascular lumen formation can be found in [52].

### Branching

Once a daughter vessel has completely matured, the epithelium may again be activated by VEGF and new sprouts can form. Sprouts are formed from the daughter vessel in the same way as they are formed from the parent vessel. The process of newly formed sprouts originating from a vessel that did not exist before tumor angiogenesis began is called branching [61, 62]. As the concentration of VEGF increases, specifically in regions in proximity to the tumor, the number of branches increases substantially due to the greater magnitude of the spatial gradient in VEGF concentration [63]. This complex network of vasculature, while leaky and tortuous [64], supplies nutrients to the tumor cells, frequently rekindling the intense proliferation that was halted by the hypoxia that initiated the entire process of angiogenesis [65].

### Anastomosis

As the branching vessels develop and migrate through the microenvironment, they anastomose with each other to establish a capillary-like network in the tumor [66]. This network, and therefore anastomosis, is paramount in delivering nutrients to the tumor microenvironment [67]. The anastomosis between tumor capillaries and the host arterioles seem random and leaves the network irregular and chaotic [64, 68], in many cases it is impossible to differentiate venules, capillaries, and arterioles [67]. Despite these abnyormalities, this network establishes blood flow and is essential for the delivery of nutrients to the tumor. Capillaries that do not form anastomosis are essentially dead ends and cannot sustain blood flow and therefore cannot effectively deliver nutrient to the tumor microenvironment [69].

## Model development

**Fig 1:**
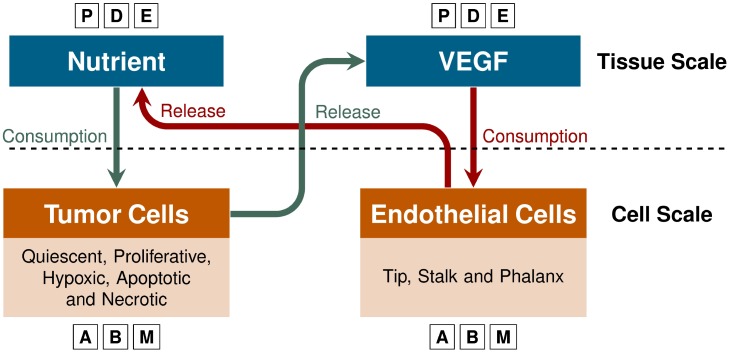
Model overview. The nutrient and VEGF dispersions that occur at the tissue scale are modeled by partial differential equations (PDEs). At the cell scale, endothelial and cancer cells are described using an agent-based model (ABM). The release and uptake of nutrient and VEGF link the PDEs and the ABM. For example, tumor cells consume nutrients and, when hypoxic conditions arise, release VEGF causing the endothelial cells to migrate up the VEGF gradient to deliver nutrients to the tumor.

We incorporate angiogenesis into an avascular, multiscale model we have previously developed [21]. At the cellular scale, an ABM describes cell division and growth, the phenotypic transitions of tumor and endothelial cells, and the movements of cells based on the balance of forces according to Newton’s second law. At the tissue scale, the reaction-diffusion equations are integrated into a continuum model that governs the VEGF and nutrient fields. Cellular actions impact the continuum model by consuming or releasing nutrients and VEGF, while the local concentration of VEGF and nutrient influence the phenotypic changes in the ABM (e.g., in favorable conditions a quiescent tumor cell will become proliferative, or a phalanx cell will become activated due to the concentration of VEGF). Phenotypic transitions according to the concentrations of VEGF and nutrient couple the agent-based and continuum models, allowing cell-to-cell interactions to compute macromolecule fields at the tissue level via finite element methods. An illustrative schematic of this model is shown in Fig 1.

### Agent-based model

We take an object-oriented approach to model each cell (both tumor and endothelial) in a data structure. This structure stores the position (***x***), velocity (***υ***) and the forces acting on each cell (e.g., drag, cell-cell, and cell-boundary forces). Additionally, the nuclear (*R_N_*), cytoplasmic (*R*), and the action (*R_A_*) radii are all stored, enabling tracking cell growth and allowing cell size variability during the simulation. As cells are not necessarily circular (in particular, endothelial cells elongate in the axial direction of the vessel [70]), a fundamental limitation of the proposed approach is that inability to capture the morphology of each individual cell. The action radius serves to partially address this limitation by allowing cell deformation within a region *R_A_* to maintain adhesion bonds, where *R_A_* > *R* [20]. Cells are free to move throughout the domain unrestrained by a grid or lattice system.

#### Cell movement

The movement of each cell is guided by the balance of the forces between the cells. This depends on cell position, time, and the characteristics of the microenvironment (e.g., the tip cell is guided by the gradient of VEGF). We assume the following forces are acting on every cell:

1. drag force (***F***_d_),
2. cell-cell adhesive (***F***_cca_) and repulsive (***F***_ccr_) forces,
3. compression (***F***_ct_) and resistance to compression (***F***_rct_) forces from the boundary.

The drag force is given by ***F***_d_ = −*η**υ***, where the constant *η* depends on the fluid viscosity [21]. The other forces acting on the cells are proportional to the adhesion (*φ*) and repulsion (*ψ*) potentials [20, 21], respectively, given by:

$$\begin{array}{ccc} \nabla \varphi & = & \left\{ \begin{array}{cc} {\left(\frac{\left|d\right|}{R_{A}}-1\right)}^{2}\frac{d}{\left|d\right|}, & 0\leq \left|d\right|\leq R_{A},\\ 0, & \text{otherwise}, \end{array}\right. \end{array}$$ (1)

$$\begin{array}{ccc} \nabla \psi & = & \left\{ \begin{array}{cc} -(\frac{R_{N}\left|d\right|}{R^{2}}-\frac{2|d|}{R}+1)\frac{d}{\left|d\right|}, & 0\leq \left|d\right|\leq R_{N},\\ -(\frac{{\left|d\right|}^{2}}{R^{2}}-\frac{2|d|}{R}+1)\frac{d}{\left|d\right|}, & R_{N}\leq \left|d\right|\leq R,\\ 0, & \text{otherwise}, \end{array}\right. \end{array}$$ (2)

where ***d*** is the distance between the center of the two cells, or the distance between the center of the cell and the boundary of the domain. Within the nuclear region, *ψ* is linear to model the increased cell stiffness in the nucleus [20]. The forces acting on i^th^ cell due to the j^th^ cell and the boundary are given as:

$$\begin{array}{c} \left\{ \begin{array}{ccc} F_{cca}^{ij} & = & -c_{cca}\nabla \varphi \left(d^{ij};R_{A}^{i}+R_{A}^{j}\right),\\ F_{ccr}^{ij} & = & -c_{ccr}\nabla \psi \left(d^{ij};R_{N}^{i}+R_{N}^{j},R^{i}+R^{j}\right),\\ F_{ct}^{i} & = & -c_{ct}K\left(N_{out},t\right)\nabla \varphi \left(d_{n}^{i};R_{A}^{i}\right);\\ F_{rct}^{i} & = & -c_{rct}K\left(N_{out},t\right)\nabla \psi \left(d_{n}^{i};R_{N}^{i},R^{i}\right), \end{array}\right. \end{array}$$ (3)

where the positive constants c_cca_, c_ccr_, c_ct_, and c_rct_ are scaling parameters, N_out_ is the number of cells that left the domain through the boundary, and K(N_out_, t) models the stiffness of the boundary. This function is bounded between zero and one and the two limiting cases can be interpreted as: 1) K(N_out_, t) = 0, there is no stress accumulation and the tumor cells can leave the domain, and 2) K(N_out_, t) = 1, the boundary behaves like a non-permeable incompressible membrane, so the tumor is compressed.

Assuming that the forces acting on the *i*^*th*^ cell equilibrate quickly, Newton’s second law yields the movement of the cell as:

0≈miυ˙i=∑j=1j≠iN(t)(Fccaij+Fccrij)+Fcti+Frcti+Fdi, (4)

where *N*(*t*) is the number of cells, and *m* the mass of the cell. Replacing the drag force (***F***_*d*_) on Eq (4), leads to

υi=1η(∑j=1j≠iN(t)(Fccaij+Fccrij)+Fcti+Frcti). (5)

Knowing the velocity of the cell from Eq (5), the position of the *i*^*th*^ cell at time *t* is given as:

$$\begin{array}{ccc} x_{i}\left(t\right) & = & x_{i}\left(t-1\right)+\upsilon_{i}\Delta t, \end{array}$$ (6)

where Δ*t* is the time interval between *t* − 1 and *t*.

#### Tumor cells

**Fig 2:**
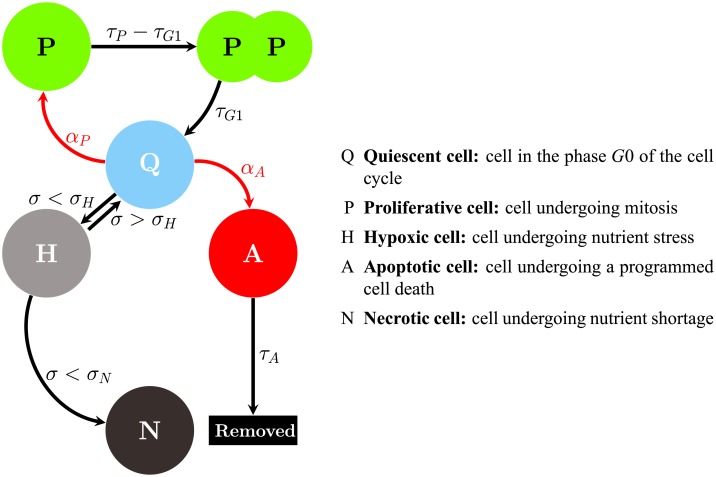
Schematic illustration of tumor cell transitions. The arrows show the potential transitions a tumor cell may undergo from each phenotype. For example, a quiescent cell (Q) may transition to a proliferative cell (P) if the local nutrients exceed a threshold *σ*_p_, to a hypoxic cell (H) if the nutrients are below a threshold *α*_H_, or to an apoptotic cell (A) if a probability *α*_A_ is met.

The adhesive and repulsive forces, movements, and phenotypic transitions of tumor cells are similar to those described in [21]. In Fig 2, we present a diagram of the phenotypic transitions of the tumor cells. Quiescent tumor cells (Q), may become proliferative (P) accordingly to a stochastic process that depends upon the proliferation function *α*_P_. The proliferative cell type divides after time *τ*_p_ − *τ*_G1_ has passed, generating two daughter cells with half of its volume (i.e., the radius of the daughter cell is the radius of the original cell divided by $\sqrt{2}$). These time values are related to the total cell cycle time (*τ*_*P*_), and the time a cell is in the G1 phase of the cell cycle (*τ*_G1_), the gap between mitosis and DNA replication [71]. The growth of the radius of these daughter cells is given by

$$\begin{array}{c} R_{\alpha }^{i}=\frac{{\bar{R}}_{\alpha }}{\sqrt{2}}\sqrt{1+\frac{\tau_{G1}+\tau -\tau_{P}}{\tau_{G1}}}, \end{array}$$ (7)

where $R_{\alpha }^{i}$ is the radius of the *i*^*th*^ cell (i.e., *R*, *R_N_* and *R_A_*), ${\bar{R}}_{\alpha }$ is the average cell radius (e.g., ${\bar{R}}_{N}$ is the average nuclear radius of a tumor cell population), and *τ* is the time of the cell in the current state. Eq (7) is a linear interpolation of the cell radii from the time that the cell begins growth, *τ* = *τ*_*P*_ − *τ*_*G*1_, to the time that it is fully grown, *τ* = *τ*_*P*_, where $R_{\alpha }^{i}={\bar{R}}_{\alpha }$. Once the radius of the daughter cell reaches the size of the average cell radius, the daughter cell becomes quiescent. The quiescent cells can go through programmed cell death and become apoptotic, at a rate, *α*_A_. Apoptotic cells are removed from the simulation after a time *τ*_A_. The transitions to the proliferative and apoptotic states are stochastic and, given that the cell is in a quiescent state Q, the probability to change to the state (*P*) or (*A*) is:

$$\begin{array}{ccc} P\left(P|Q\right)=1-\text{exp}(-\alpha_{P}\Delta_{t}), & \text{where} & \;\alpha_{P}\left(t\right)={\bar{\alpha }}_{P}(\frac{\sigma -\sigma_{H}}{1-\sigma_{H}}), \end{array}$$ (8)

$$\begin{array}{ccc} P\left(A|Q\right)=1-\text{exp}(-\alpha_{A}\Delta_{t}), & \text{where} & \;\alpha_{A}=\text{constant}, \end{array}$$ (9)

where the constant ${\bar{\alpha }}_{\text{p}}$ is the maximum probability of a cell to transition from quiescent to proliferative (when *σ* = 1, *α* = *α*_*P*_), *σ* is the normalized concentration of nutrient, and *σ*_H_ is the threshold for a cell to transition to hypoxic [20, 21].

During the maintenance of cellular function, nutrients are consumed by proliferative, quiescent, and hypoxic tumor cells. The rate of consumption is determined by the continuum model description (see Continuum Model section). After substantial depletion of nutrients, quiescent cells become hypoxic when the local value of nutrients is not sufficient to allow tumor cells to maintain proper cellular function. This threshold is noted as *σ*_H_. Hypoxic cells are crucial for the process of angiogenesis; in particular, it has been shown that hypoxic cells are primarily responsible for the release of VEGF [72]. If the local nutrient becomes less than *σ*_N_, the cell becomes necrotic. The nutrient concentration can increase due to the new vasculature and, if the nutrient level becomes favorable for tumor growth (i.e., *σ* ≥ *σ*_H_), hypoxic cells can return to the quiescent state. Necrotic, or dead cells, can no longer be revived by new nutrients supplied to the tumor.

#### Endothelial cells

**Fig 3:**
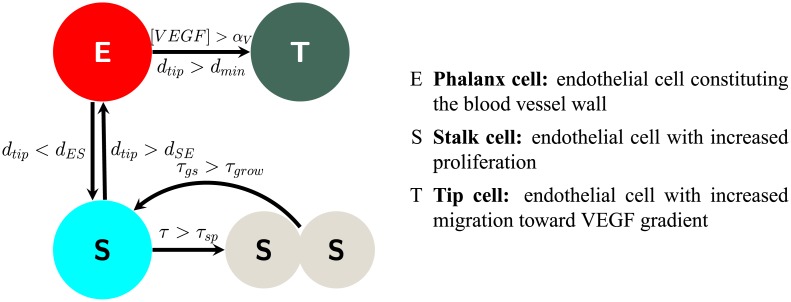
Schematic illustration of endothelial cell transitions. The arrows show the transitions that each endothelial cell phenotype may experience. Phalanx cells (E) may transition to tip cells if the concentration of VEGF is greater than a threshold *α*_V_ and is greater than d_ES_ away from the closest tip cell (T) or to a stalk cell (S) if the distance to an activated tip cell is less than d_SE_. Stalk cells may divide after time *τ*_SP_ and then have a growing period *τ*_gs_.

Our ABM approach to modeling angiogenesis incorporates the “tip-stalk-phalanx” concept, described in the Biological Background section. We assume that the VEGF released by the tumor cells is the dominating factor in promoting angiogenesis. To capture the heterogeneity of the endothelial cells during angiogenesis, we have identified rules to govern phenotypic transitions. The phenotypes selected to model sprouting are the migratory tip cell (T), a proliferative stalk cell (S), and a phalanx cell (E) [50, 52], as shown in Fig 3. Activation of phalanx cells occurs when the normalized concentration of VEGF becomes greater than the threshold, *α*_V_. However, due to competition between activated endothelial cells, a distance parameter (d_tip_) is imposed upon the selection of new tip cells. The formulation for competition is implemented as follows: if a phalanx cell E_i_ is activated due to the concentration of VEGF, [VEGF], rising above a threshold, but there remains a tip cell T_j_ such that the distance between E_i_ and T_j_, (d_tip_), is less than a minimum distance, (d_min_), then E_i_ remains a phalanx cell. However, if [*V E G F*] > *α*_V_ and *d*_tip_(*E*_i_, *T*_j_}) > *d*_min_, E_i_ becomes an activated tip cell T_i_. A limitation of this formulation is that only phalanx cells can transition to tip cells, though it is known that stalk cells compete for the tip cell phenotype and may transition to a tip cell [73]. Incorporating this would add more rules governing phenotypic transitions and computational complexity, which we aim to minimize, and would have a negligible effect on the vessels produced in simulations (the characteristic length scale between tip and stalk cells is on the order of 10*μm* while the length of the vessels is an order of magnitude greater).

Newly activated tip cells search the nearby area and change phalanx cells into stalk cells. To become a stalk cell, the distance between the phalanx cell and the new tip cell, d_tip_, must be less than a minimum distance, d_ES_. However, a stalk cell can return to its previous state, a phalanx cell, if d_tip_ becomes greater than d_ES_. After the phenotypic transition from phalanx to stalk, the activated tip cell will no longer change the phenotypes of nearby phalanx cells. New stalk cells are only developed afterward by mitosis.

#### Tip cell dynamics

The movement and guidance of sprouting blood vessels is controlled by the tip cell. To mimic the movement of the tip cell due to chemical signaling, we introduce a force ***F***_VEGF_. The directionality of the force is according to the gradient of VEGF, given as

$$\begin{array}{ccc} F_{VEGF}^{ij} & = & -c_{tm}\nabla \bar{\psi }\left(d^{ij};R_{N}^{i}+R_{N}^{j},R^{i}+R^{j},\left[VEGF\right]\right), \end{array}$$ (10)

where *c*_*tm*_ is the scaling parameter with units of kg · m · μm/s^2^, ***d***^*ij*^ is the distance between the tip cell *i* and stalk cell *j*, and the VEGF potential [20, 21], $\bar{\psi }$, is given as

$$\begin{array}{ccc} \nabla \bar{\psi }\left(d;R_{N},R,\left[VEGF\right]\right) & = & \left\{ \begin{array}{cc} -(\frac{R_{N}\left|d\right|}{R^{2}}-\frac{2|d|}{R}+1)\frac{\nabla [VEGF]}{\left|\nabla [VEGF]||d\right|}, & 0\leq \left|d\right|\leq R_{N},\\ -(\frac{{\left|d\right|}^{2}}{R^{2}}-\frac{2|d|}{R}+1)\frac{\nabla [VEGF]}{\left|\nabla [VEGF]||d\right|}, & R_{N}\leq \left|d\right|\leq R,\\ 0, & \text{otherwise}. \end{array}\right. \end{array}$$ (11)

**Fig 4:**
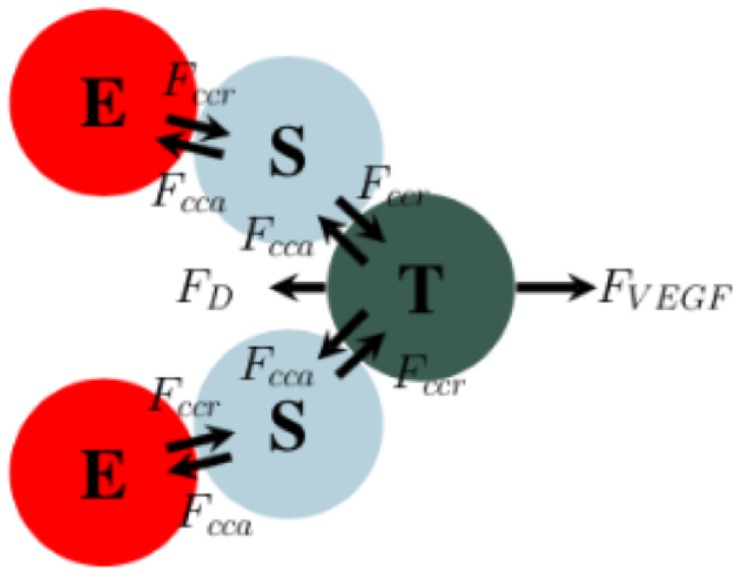
Endothelial cell forces. F_VEGF_ is the chemotactic force due to the gradient of VEGF. F_cca_ and ***F***_*ccr*_ are the cell-cell adhesive and repulsive forces, respectively. F_d_ is the drag force associated with the extracellular matrix. The balance between adhesion and repulsion forces acting on the cells is responsible for maintaining the vessel integrity. The chemotactic force acting on the tip cell drives the movement of the tip, which, by the interaction with other cells, guides the remodeling of the vessel. (E = phalanx cell, S = stalk cell, and T = tip cell).

This new force is added to the balance of the forces (Eq (4)) acting on the tip cells. While the tip cell is moving along the gradient of VEGF, due to the balance of this force and the adhesion and repulsion forces between tip and stalk cells, it is responsible for slight remodeling of the new sprout. To safeguard the sprout from breaking due to high gradients in the VEGF field (i.e., tip cells moving freely without being connected to the sprout), the potential function defined by Eq (11) is proportional to the distance to the stalk cells surrounding the tip cells. This function, regardless of the gradient of VEGF, acts to scale the force so that it does not dominate the adhesion forces of the tip and stalk cells. A diagram of a sprout is shown in Fig 4.

A delicate balance of forces is required for modeling the development of vessel sprouts. The interplay between the adhesion and repulsion forces and the VEGF force results in the overall geometry of the vessel. If ***F***_VEGF_ is the dominant factor, the vessel is guided by the tip cell “dragging” the vessel from the front. Similarly, if the adhesion force between the tip and stalk cells is the dominant factor, the vessel is guided primarily by the proliferation of the stalk cells. The velocity of the tip cell is obtained similarly as presented in Eq (5), with the inclusion of the ***F***_VEGF_ force.

In addition to these forces, we also include a force that promotes vessel anastomosis. Tip cells will search in the direction of the gradient of VEGF to see if another vessel intersects its path. If the tip cell finds another vessel it will continue to migrate in the direction of this VEGF gradient, and will no longer sample the microenvironment for an updated VEGF gradient. Tip cells fuse with another vessel if the distance requirement of the intersecting vessel is less than or equal to *d*_*ths*_, given in Eq (12) as:

$$\begin{array}{c} d_{ths}=2.25\cdot min\left(cell_{a}.R,cell_{b}.R\right) \end{array}$$ (12)

where *d*_ths_ is the distance threshold between the anastomosing tip cell and the endothelial cells of the vessel wall and *cell*_i_.*R* is the cytoplasm radius of cell *i*. This threshold is chosen so that the stalk cells behind the tip cell do not interact with the anastomosing vessel, as they could repel each other and inhibit anastomosis (see Lumen Formation section).

#### Lumen formation

**Fig 5:**
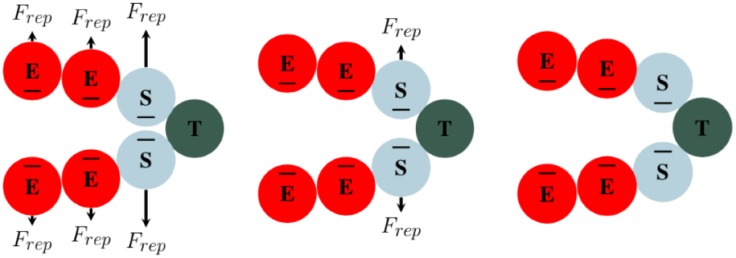
Time evolution of lumen formation. Blood vessel walls are polarized (shown as a negative charge (-) on the apical membrane of the endothelial cells), leading to a repulsive force that separates the vessel. On the left, the phalanx (E) and stalk (S) cells are close together, causing a large repulsion force to expand the lumen. In the middle, the phalanx cells are now in equilibrium; however, the stalk cells still repel each other. On the right, the blood vessel is in equilibrium and there is no longer any repulsion acting on the cells.

To accurately model angiogenesis and the shape of the vascular network, the diameter of the involved blood vessels is a crucial parameter [74]. Here, the lumen formation is modeled by a repulsive force between endothelial cells on opposite sides of the blood vessel wall (based on the cell-cell repulsion method described in Lumen Formation section). To distinguish between sides of the blood vessel wall, we introduce a cell characteristic termed *previous state*. To form the lumen, both sides of the wall are taken to be polarized and are, therefore, repelling one another. The repulsion force acting on the lumen is proportional to Eq (2), and given as:

$$\begin{array}{ccc} F_{rep}^{ij} & = & -c_{rep}\nabla \psi \left(d^{ij};R_{N}^{i}+R_{N}^{j},R_{rep}^{i}+R_{rep}^{j}\right), \end{array}$$ (13)

where the positive constant *c*_*rep*_ is a scaling parameter, the *i*^*th*^ and *j*^*th*^ cells are in opposite side of the vessel, and *R*_*rep*_ is the repulsion distance. The distance R_rep_ governs the diameter of the lumen directly behind the tip cell; thus, it is constrained by the action radius of the tip and stalk cells. If chosen too large (i.e., *R*_rep_ >> *R*_A_), the stalk cells would not be within the adhesion distance from the tip cell and the vessel would break. Here, we take the coefficient of repulsion, c_rep_, such that F_rep_ is greater the adhesion forces acting on the lumen. These constraints, *F*_rep_ > *F*_cca_, and the choice of R_rep_, maintain the aesthetics of the lumen and avoid the collapse of the vessel without external forces (note the vessel can still collapse when compressed by the tumor). The lumen repulsion dynamics are shown in Fig 5.

#### Stalk cell dynamics

Once a tip cell is selected and local phalanx cells transition into stalk cells, the sprout is guided according to the adhesion and repulsion on the tip cell and the gradient of VEGF. However, the elongation of the forming sprout is due to the continued proliferation of the stalk cells directly behind the tip cell. Stalk cell division occurs deterministically after time, t_sp_, has passed since the stalk cell reached its full size. After stalk cell division, two daughter stalk cells are produced. The radii of the daughter cells are such that the area of each cell is half of the parent cell. The new location of the daughter cells (***x***_d1_ and ***x***_d2_) are aligned with the locations of the tip cell (***x***_t_) and the parent stalk cell (***x***_s_), such that

$$\begin{array}{c} x_{d1}=x_{s}+0.25\left(x_{t}-x_{s}\right), \end{array}$$ (14)

$$\begin{array}{c} x_{d2}=x_{s}-0.25\left(x_{t}-x_{s}\right). \end{array}$$ (15)

**Fig 6:**
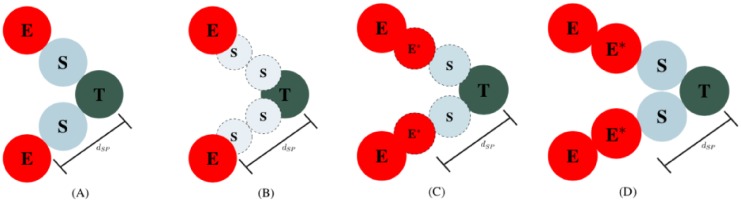
Stalk cell division. Panel A depicts initial sprouting with both stalk (S) cells fully matured, while Panel B shows the initial placement of the daughter cells. Panel C depicts how the combination of continued stalk cell proliferation and migration of the tip cell due to the VEGF gradient leads to elongation of the sprout. The stalk cell phenotype is governed by complex signaling pathways and local variations in the VEGF field [52]. Since these pathways (primarily the notch signaling pathway) are contact dependent, the stalk cell phenotype is fundamentally limited by its distance to a tip cell. We simplify this process by implementing a distance threshold d_SE_, the distance from a tip cell where a stalk cell will transition to a phalanx cell. Finally, Panel D displays that the growing stalk and phalanx cells are fully matured, and the process restarts again from Panel A. This process is continued repeatedly to allow sprout elongation in the direction of the gradient of VEGF.

Once proliferation occurs, the nucleus and cytoplasmic radius of daughter cells begin to grow according to:

$$\begin{array}{c} R_{\alpha }^{i}=\frac{{\bar{R}}_{\alpha }^{e}}{\sqrt{2}}\sqrt{1+\frac{\tau_{G1}^{e}+\tau -\tau_{P}^{e}}{\tau_{G1}^{e}}}, \end{array}$$ (16)

where $\tau_{P}^{e}$ is the total cell cycle time for endothelial cells, $\tau_{G1}^{e}$ is the time an endothelial cell is in the G1 phase of the cell cycle, $R_{\alpha }^{i}$ is the radius of the *i*^*th*^ cell (i.e., nucleus and cytoplasm radius), ${\bar{R}}_{\alpha }^{e}$ is the average endothelial cell radius, and *τ* is the time of the cell in the current state. However, the action radius of the stalk cell does not change after proliferation or during growth. We assume that the small difference in size between the daughter and parent cell does not influence the maximum adhesion distance between cells. A graphic of stalk cell proliferation is shown in Fig 6.

#### Branching

**Fig 7:**
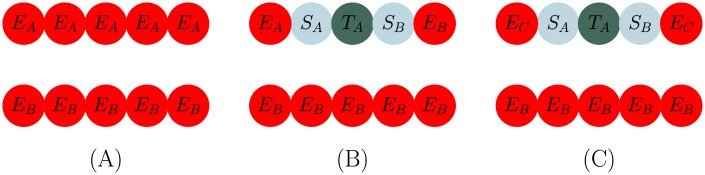
Schematic of vessel branching. Panel A depicts the initial blood vessel made up of wall *X* (comprised of cells E_*A*_) and wall *Y* (comprised of cells E_*B*_). In Panel B, a tip cell (T_*A*_) is selected on vessel wall X; however, applying the basic rules for a new tip cell selection, stalk cell S_*B*_ would repel against adjacent phalanx cell E_*A*_ causing the vessel to break. For this reason, we implement a previous state C which acts as a reset and allows the newly transitioned cell S_*B*_ to adhere to other cells in wall *X*, shown in Panel C.

The criteria for tip cell selection during branching remains the same as in the Endothelial Cell section (i.e. [VEGF] is greater than a threshold and the distance to another tip cell is above a threshold). However, due to repulsion between endothelial cells with different previous states, adjustments to the process must be made. For a 2D example, shown in Fig 7A, consider two sides of the blood vessel wall, *X* (comprised of cells E_*A*_) and *Y* (comprised of cells E_*B*_), where subscripts *A* and *B* are the cells previous state. Repulsion between these two walls remains, even if a phalanx cell E_*A*_ on wall *X* transitions into a tip cell. However, upon becoming a tip cell, cell E_*A*_ (now labelled as T_*A*_, as it is now a tip cell) changes adjacent phalanx cells to stalk cells S_*A*_ and S_*B*_ (see Fig 7B). The previous state (which distinguishes repulsion between cells) of these new stalk cells become *A* and *B*. Since all the cells on wall *X* have previous state *A*, except for stalk cell S_*B*_ (which is now previous state *B*), the repulsion between S_*B*_ and all other endothelial cells (E_*A*_), would break the vessel. To allow for branching, we implement a “previous state reset”. While changing the phalanx cells on each side of the tip cell to stalk cells, the activated tip cell also resets the previous state of the phalanx cell on the other side of the new stalk cell to previous state *C*, which adheres to both previous state *A* and *B* cells, depicted in Fig 7C.

If the distance between two tip cells becomes less than a threshold d_TT_ (i.e., the two tip cells have fused together and formed an anastomosis), both cells transition back to phalanx cells and reset their previous state, allowing for tip cell selection to repeat based on local concentrations of VEGF. This mechanism allows for sprouting vessels to form an anastomosis through tip cell fusion and new competition among the surrounding endothelial cells. After the initial period T_tip-p_, the interaction between the tip and phalanx cells is governed by the balance of adhesion and repulsion forces, F_cca_ and F_ccr_, respectively. If the tip is in close proximity to another sprout or parent vessel, the adhesion between tip and phalanx cells promotes anastomosis within the model and the tip cell transitions back into a phalanx cell. After daughter vessels anastomose together, they begin to mature and establish blood flow through the vascular network. Since we do not explicitly model blood flow and use endothelial cells as a surrogate to deliver nutrients, we impose the restriction that endothelial cells must be part of a looped vessel to release nutrient. This is accomplished by flagging cells that spearhead the anastomosis between two vessels and iterate through all cells along the vessel wall in between them. If all these cells are connected (they may become disconnected by proliferating tumor cells), they begin to release nutrient. If the vessel is sectioned off and any cells between the two flagged cells become deactivated, the vessel loop is broken and all of these cells stop releasing nutrient.

During vascular tumor growth, the newly formed vascular network increases the nutrient availability around the tumor. At this stage, the tumor continues to grow, which may lead it to surround the vessels. With the increasing number of tumor cells around the vessel, the forces F_cca_ and F_ccr_ acting on the phalanx cells can overcome the repulsion force, F_rep_, responsible for lumen integrity. If the compression acting on the vessel is higher than F_rep_, the vessel collapses its walls, breaking the adhesion between neighboring cells and is sectioned off from the parent vessel. In cases where the vessel is sectioned off from the parent vessel, the cells making up the sectioned vessel are deactivated. These deactivated cells are flagged so that they cannot be selected to be a tip cell again.

### Continuum model

As described above, the transitions between different phenotypes (e.g., proliferative, hypoxic and necrotic tumor cells, and tip-stalk-phalanx endothelial cells) are dependent on the nutrient and VEGF concentrations available in the microenvironment, *σ* and [VEGF], respectively. The dispersion of these two concentrations are modeled at the tissue scale, as they are taken to be heterogeneous fields that can freely diffuse throughout the domain. The models are derived from continuum physics assuming conservation of mass. The mass balance principle yields models based on reaction-diffusion equations, where the bridge between tissue and cell scales happens through the source and reaction terms.

We consider the endothelial cells as a surrogate for blood flow as they are responsible for the delivery of nutrients. To avoid the delivery of nutrients by endothelial cells that are part of vessels severed from the parent vessel, only endothelial cells that are part of anastomotic loops (as described in the Biological Background) “deliver” nutrients. The nutrients are consumed by tumor cells, with the exception of non-viable cells (i.e., dying and necrotic cells). When the local nutrient level falls below the threshold *σ*_H_, tumor cells become hypoxic and release VEGF to trigger the growth of new blood vessels. During this process, VEGF is consumed, decreasing the available concentration in the environment. With these assumptions, the nutrient and VEGF concentrations, (*σ*(***x***, *t*)) and ([*VEGF*](***x***, *t*)), respectively, are governed by the following reaction-diffusion equations:

$$\begin{array}{c} \begin{array}{ccc} \frac{\partial \sigma }{\partial t} & = & \nabla \cdot \left(D_{n}\nabla \sigma \right)-\Lambda_{n}\left(x,t\right)\sigma +\Gamma_{n}\left(x,t\right)\sigma \left(1-\sigma \right),\\ \frac{\partial [VEGF]}{\partial t} & = & \nabla \cdot \left(D_{v}\nabla \left[VEGF\right]\right)-\Lambda_{v}\left(x,t\right)\left[VEGF\right]\\ & + & \Gamma_{v}\left(x,t\right)\left[VEGF\right]\left(1-\left[VEGF\right]\right), \end{array}\}\;\text{in}\;\Omega \times \left(0,T_{tissue}\right), \end{array}$$ (17)

where D_n_ and D_v_ are the nutrient and VEGF diffusion coefficients, respectively, Λ_n_(***x***, *t*) is the nutrient uptake rate of the cancer cells, Λ_v_(***x***, *t*) is the VEGF uptake rate of the endothelial cells, Γ_*n*_(***x***, *t*) is the nutrient delivery rate of looped endothelial cells, and Γ_v_(***x***, *t*) is the VEGF release rate of hypoxic cells. The release of nutrient and VEGF are modeled by a logistic production term, so their concentration is bounded between zero and one. The system is assumed to be isolated; i.e., no flux through the boundary, leading to the application of the following Neumann boundary condition:

$$\begin{array}{c} \nabla \sigma \cdot n=\nabla \left[VEGF\right]\cdot n=0,\;\text{on}\;\partial \Omega \times \left(0,T_{tissue}\right), \end{array}$$ (18)

where *n* is a unit exterior normal vector on the boundary ∂Ω.

In Eq (17), the functions Λ_n_, Γ_n_, Λ_v_, and Γ_v_, are the nutrient uptake by tumor cells, nutrient delivery by looped endothelial cells, VEGF uptake by endothelial cells, and VEGF release by hypoxic tumor cells, respectively. These functions serve to bridge the tissue and cell scales by capturing the production and consumption of VEGF and nutrient by the cells, and act as a source or sink term in the continuum model that governs the tissue scale. The functions average the cell scale volume fractions of cells in each element of the finite element mesh and produce or consume VEGF and nutrient at the element nodes. These functions are defined as:

$$\begin{array}{c} \begin{array}{ccc} \Lambda_{n}\left(x,t\right) & = & \lambda_{pq}^{c}\phi_{pq}\left(x,t\right)+\lambda_{h}^{c}\phi_{h}\left(x,t\right)+\lambda_{decay}^{\sigma }(1-\phi_{c}\left(x,t\right)),\\ \Gamma_{n}\left(x,t\right) & = & \gamma_{e}\phi_{e}\left(x,t\right),\\ \Lambda_{v}\left(x,t\right) & = & \lambda_{t}^{c}\phi_{t}\left(x,t\right)+\lambda_{s}^{c}\phi_{s}\left(x,t\right)+\lambda_{p}^{c}\phi_{p}\left(x,t\right)+\lambda_{decay}^{VEGF}(1-\phi_{c}\left(x,t\right)),\\ \Gamma_{v}\left(x,t\right) & = & \gamma_{h}\phi_{h}\left(x,t\right), \end{array}\} \end{array}$$ (19)

where the subscripts *pq*, *h*, *e*, *t*, *s*, *p*, and *c* indicate proliferative plus quiescent tumor cells, hypoxic cells, looped endothelial cells, tip cells, stalk cells, phalanx cells, and all (tumor and endothelial) cells, respectively. In Eq (19), *ϕ*_*α*_(***x***, *t*) is the volume fraction of the cell *α*, *α* ∈ {*pq*, *h*, *e*, *t*, *s*, *p*, *c*}, at position *x* and time *t*, $\lambda_{\alpha }^{c}$ is the consumption rate by the *α* cell, *γ*_*α*_ is the production rate of the *α* cell, and $\lambda_{decay}^{\sigma }$ and $\lambda_{decay}^{VEGF}$ are the natural decay of the nutrient and VEGF, respectively. See Table 1 below for a complete listing of all model parameters and their definitions.

### Sensitivity analysis

The objective of a sensitivity analysis is to quantify the contributions of model parameters to the uncertainty in the model output [75]. In this work, we employ a variance-based global sensitivity analysis method to quantify the contributions of the parameters given in Eq (17). The variance-based method, also known as the Sobol method [76, 77], is a rigorous global method that takes into account both first order parameter effects (i.e., single parameter effect on the model output), and higher order effects (i.e., the effect of parameter to parameter interactions on the model output). Here, we employ the sampling strategy and estimator recommended in [78]. The computational cost of the analysis is dependent on the number of parameters, *k*, and the sample size, *N*, with the total number of model evaluations given as *N*_T_ = *N*(*k* + 1).

We utilize the radial sampling algorithm, which consists of generating two matrices, ***A*** and ***B***, with size *N* × *k*. The next step is to generate *k* matrices $A_{B}^{\left(k\right)}$, where the column *k* comes from ***B*** and all other columns are from ***A***. To illustrate the generation of these matrices, we present the matrices for the case where *N* = 1:

$$\begin{array}{cccccccccc} A & =[a_{1,1}\; & & a_{1,2}\; & & \ldots \; & & a_{1,k} & & ],\\ B & =[b_{1,1} & & b_{1,2} & & \ldots & & b_{1,k} & & ],\\ A_{B}^{\left(1\right)} & =[b_{1,1} & & a_{1,2} & & \ldots & & a_{1,k} & & ],\\ A_{B}^{\left(2\right)} & =[a_{1,1} & & b_{1,2} & & \ldots & & a_{1,k} & & ],\\ \vdots \\ A_{B}^{\left(k\right)} & =[a_{1,1} & & a_{1,2} & & \ldots & & b_{1,k} & & ]. \end{array}$$

For each row of each matrix (i.e., **A** and $A_{B}^{k}$), which represents one set of values for the vector of parameters, we compute the output of the model (the number of each cell phenotype). These outputs are stored as vectors *Y*_*A*_ and $Y_{AB}^{k}$. With these vectors, we compute the total effect index (TEI), $S_{T_{i}}$, for each parameter *i* [79]. For non-additive models (i.e., when it is impossible to isolate the effects of its parameters in a variance decomposition framework; e.g, *Y* = π*_i_Z_i_* [77]), the total effect index takes into account both the first-order effects and the contribution of higher-order effects due to interactions between the model parameters. We compute $S_{T_{i}}$ as:

$$\begin{array}{c} S_{T_{i}}=\frac{1}{2N}\sum_{j=1}^{N}{\left({\left(Y_{A}\right)}_{j}-{\left(Y_{AB}^{\left(i\right)}\right)}_{j}\right)}^{2}. \end{array}$$ (20)

A necessary and sufficient condition for the parameter to be considered noninfluential is that Eq (20) is equal to zero [77]. Thus, if $S_{T_{i}}<\epsilon$ (where *ϵ* is small relative to other $S_{T_{j}}$ and is problem dependent) then the parameter can be fixed to any value within the uncertainty range [77]. According to [77], the approximation error affecting the output of the model when the parameter *i* is fixed depends on the value of $S_{T_{j}}$.

## Numerical experiments

The ABM is implemented in C++ using an object-oriented approach (https://github.com/CalebPhillips5/ABM_Ang). The continuum model is solved using libMesh [80], a general-purpose C++ finite-element library. The graphics displaying the ABM are generated in MATLAB (The MathWorks, Natick, MA, USA) while the continuum model graphics are generated in ParaView [81]. The ABM results are generated in MATLAB due to the speed of image generation and compatibility with a variety of operating systems. ParaView is used for the continuum model because the software is compatible with supercomputing platforms and enables visualization of finite element domains.

**Fig 8:**
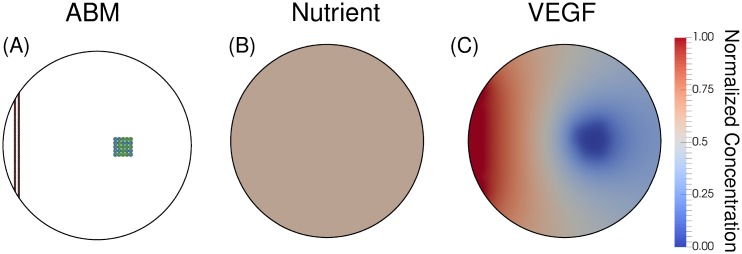
Initial conditions. Panel A displays the ABM with the parent blood vessel in red and tumor cells divided into quiescent (blue) and proliferative (green) cells. In Panels B and C, a uniform nutrient field with a normalized concentration of 0.6, and a uniform VEGF field set to zero (as the model starts without hypoxic cells), respectively. This initial condition represents a single vessel as a source of nutrients.

The parameter values for the ABM are given in Table 1, and are used throughout all simulations (unless otherwise specified for the sensitivity analysis). The computational domain is circular and measures 1000 microns in diameter. A wall of endothelial cells on the left of the domain simulate an existing blood vessel before tumor formation (see Fig 8). The tumor is comprised of 25 cells at the initial time point of each simulation, with 13 being proliferative and 12 being quiescent. The nutrient and VEGF fields are modeled as normalized concentrations, and we assume a uniform initial condition given as *σ*(*x*, 0) = 0.6 and [*VEGF*](*x*, 0) = 0.0 in Ω.

**Table 1:** Baseline set of model parameter values.

Parameter	Meaning	Value	Ref.
*R*	tumor cell radius	9.953	[20]
*R*_*N*_	tumor cell nuclear radius	5.295	[20, 82]
*R*_*A*_	tumor action radius	1.214*R*	[20, 21]
*R*^*e*^	endothelial cell radius	0.5*R*	estimated
$R_{N}^{e}$	endothelial cell nuclear radius	0.5*R*_*N*_	estimated
$R_{A}^{e}$	endothelial action radius	0.5*R*_*A*_	estimated
*c*_*cca*_	tumor cell-cell adhesion coefficient	0	estimated
*c*_*cca*_	endothelial-endothelial cell adhesion coefficient	0.488836	[20]
*c*_*ccr*_	tumor cell-cell repulsion coefficient	10	[21]
*c*_*ct*_	cell-boundary adhesion coefficient	10	estimated
*c*_*rct*_	cell-boundary repulsion coefficient	0	estimated
*τ*_*P*_	total cell cycle time	18*h*	[20, 21]
*τ*_*G*1_	G1 phase time	9*h*	[83]
*τ*_*A*_	apoptosis time	8.6*h*	[20, 21]
*τ*_*NL*_	lysing time	6*h*	[20, 21]
*τ*_*C*_	necrosis time	360*h*	[20, 21]
${\bar{\alpha }}_{P}$	proliferation intensity	0.27067 *h*^−1^	estimated
*α*_*A*_	Q→A transition intensity	0.0012728 *h*^−1^	[20, 21]
*σ*_*H*_	hypoxic threshold	0.3	estimated
*σ*_*N*_	necrotic threshold	0.25*σ*_*H*_	estimated
*α*_*V*_	VEGF threshold	0.1	estimated
*d*_*min*_	minimum distance from tip cell for new tip cell selection	8*R*_*A*_	estimated
*d*_*SE*_	minimum distance from tip cell for S → E transition	1.55*R*	estimated
*d*_*ES*_	maximum distance from tip cell for E → S transition	1.55*R*	estimated
*τ*_*grow*_	stalk cell growth time	38*h*	estimated
*c*_*tm*_	VEGF coefficient	0.6	estimated
*c*_*rep*_	lumen repulsion coefficient	1.0	estimated
*d*_*TT*_	maximum distance for tip cells to deactivate	3$R_{A}^{e}$	estimated
*D*_*n*_	nutrient diffusion coefficient	1000 μm^2^/*h*	[20, 21]
*D*_*V*_	VEGF diffusion coefficient	8000 μm^2^/*h*	estimated
$\lambda_{pq}^{c},\lambda_{h}^{c}$	nutrient consumption rate of proliferative/quiescent, hypoxic cells	0.8 *h*^−1^	[20, 21]
*γ*_*e*_	nutrient production rate of endothelial cells	300 *h*^−1^	estimated
$\lambda_{t}^{c},\lambda_{s}^{c},\lambda_{p}^{c}$	VEGF consumption rate of tip, stalk, and phalanx cells	10 *h*^−1^	estimated
*γ*_*h*_	VEGF production rate of hypoxic cells	300 *h*^−1^	estimated
$\lambda_{decay}^{VEGF}$	natural decay rate of VEGF	0.0 *h*^−1^	estimated
$\lambda_{decay}^{\sigma }$	natural decay rate of nutrient	0.0 *h*^−1^	estimated

### ABM simulations

**Fig 9:**
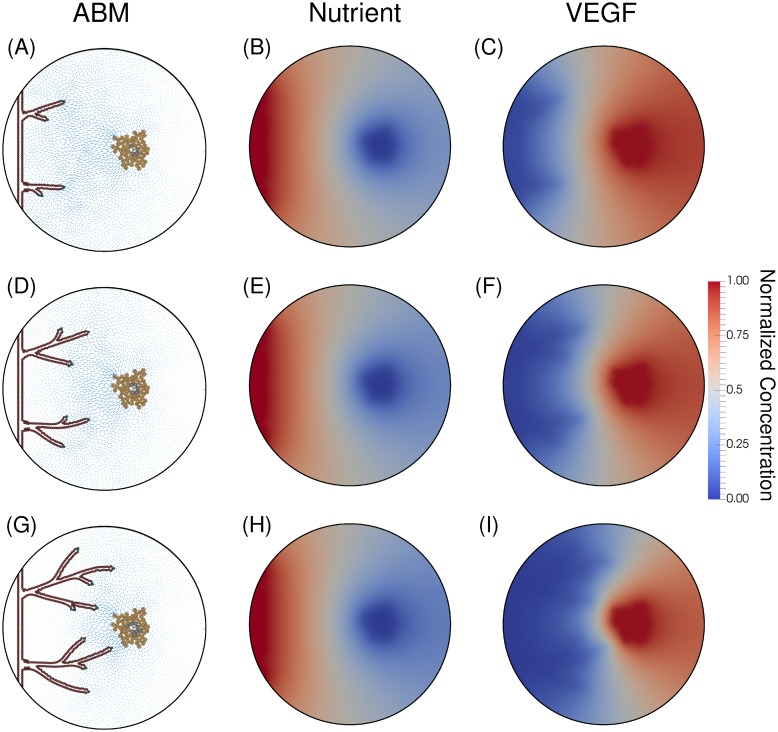
Initial angiogenic sprouting. Angiogenic sprouting occurs at hours 1500 (Panels A-C), 2000 (Panels D-F), and 2500 (Panels G-I). The left column shows the ABM with tumor cells on the right (orange = hypoxic, grey = necrotic). The blood vessels are dynamic and evolve over time with tip cells guiding each branch (green), necrotic cells just behind the tip cells (cyan), and phalanx cells establishing the lumen and maintaining the structure of the vessel (red). The arrows point in the direction of gradient of VEGF and the size of the arrow corresponds to the magnitude of the gradient. The middle column shows a relatively constant nutrient field, since vessels must form anastomotic loops in order to establish blood flow and ultimately deliver nutrients to the tumor. The final column shows the VEGF field; outlines of the vessels can be seen in shaded blue, since this region of lowest concentration of VEGF corresponds to where phalanx cells uptake VEGF released by hypoxic tumor cells.

As the tumor cells rapidly grow, they deplete the surrounding nutrients and become hypoxic. These hypoxic tumor cells release VEGF (according to Eq (17)), and trigger angiogenic sprouting on the parent vessel. As the hypoxic tumor cells continue signaling, the vessel’s branching increases, and the vascular structure becomes a complex network. The early stages of this simulated network are shown in Fig 9.

In Fig 9, Panel A depicts newly branched vessels in the presence of high concentrations of VEGF (Panel C). These vessels deplete the VEGF supply, shown in the dark blue regions of Panel C, and do not allow the formation of other new sprouts from the parent vessel. As these vessels migrate toward the tumor, depicted in Panel D, they enter a region with high concentrations of VEGF and more sprouting occurs (Panel G). Proximal to the tumor, the main driver of branching becomes the distance threshold between tip cells, since the concentration of VEGF is high enough to activate many endothelial cells. The oxygen fields (Panels B, E, and H) are relatively static because the vascular structure has not yet created loops capable of sustaining blood flow and delivering nutrients.

**Fig 10:**
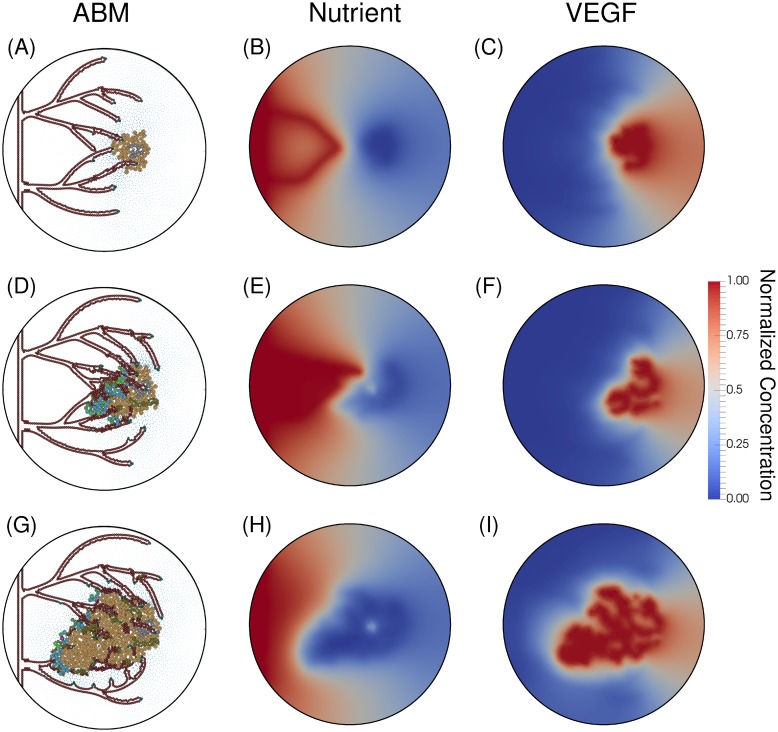
Anastomotic loop formation. The first anastomotic loop is formed at hour 3100 (Panels A-C). Panel A depicts a newly anastomosed vessel and Panel B the subsequent influx of nutrient delivered by the vessel. The tip cell of the top anastomosed vessel fused into the cells on the wall of the bottom anastomosed vessel (as dictated by the process described in the Model Development Branching subsection). At hour 3800 (Panels D-F), the tumor has become proliferative because of the supplied nutrients from the vessels (Panel E). The complexity of this vascular system is depicted in Panel E, with numerous junctures of vessels and the corresponding nutrients supplied by these vessels. With the decreasing number of hypoxic cells and the increase in vasculature, the concentration of VEGF, shown in Panel F, is quite depleted. Panel G (hour 4000) shows the resulting tumor and vasculature after the tumor has rapidly proliferated due to the influx of nutrients. The stresses imparted on the vasculature by the proliferating tumor cells has severed most of the vessels proximal to the tumor. The resulting nutrient field (Panel H) has no vessels releasing nutrients, as all anastomosed loops have been severed. With the reduction of supplied nutrients, the tumor becomes hypoxic and the production of VEGF increases again (Panel I).

In Fig 10, we present the evolution of the ABM after the anastomosis occurs. Over time the vessel network matures (Panels A, D, and G), grows toward the tumor directed by the concentration of VEGF (Panels C, F, and I), forms complex networks and anastomotic loops, and ultimately delivers nutrients to the tumor (Panels B, E, and H). In Panels A-C, we show the ability of the mathematical model to recapitulate the progression of tumor angiogenesis. Panel A depicts the first anastomosis loop, which allows the vasculature to release nutrients (Panel B). This vasculature grows and forms a network capable of sustaining blood flow through anastomotic loops (Panel D) and delivering nutrients to the tumor (Panel E). The increase of nutrient supply restarts the tumor growth (Panel D). With the rapid tumor growth, the vessels are occluded (Panel G), leading to a decrease of nutrient concentration (Panel H). Moreover, the lack of nutrients increases the number of hypoxic cells, which release VEGF (Panel I).

### Sensitivity analysis of the reaction-diffusion equations

To better understand how the parameters of the continuous model contribute to the number of each cell phenotype, we perform a sensitivity analysis on the diffusion, consumption, and production rates for the nutrient and VEGF equations as described by Eq (17), respectively. The sample size considered is *N* = 1000 and the number of parameters is *k* = 6, which are {*N*_d_ = nutrient diffusion, *N*_p_ = nutrient production, *N*_c_ = nutrient consumption, *V*_d_ = VEGF diffusion, *V*_p_ = VEGF production, *V*_c_ = VEGF consumption}. Thus, the number of model evaluation required to obtain the total effect index is *N*_T_ = 8000.

**Fig 11:**
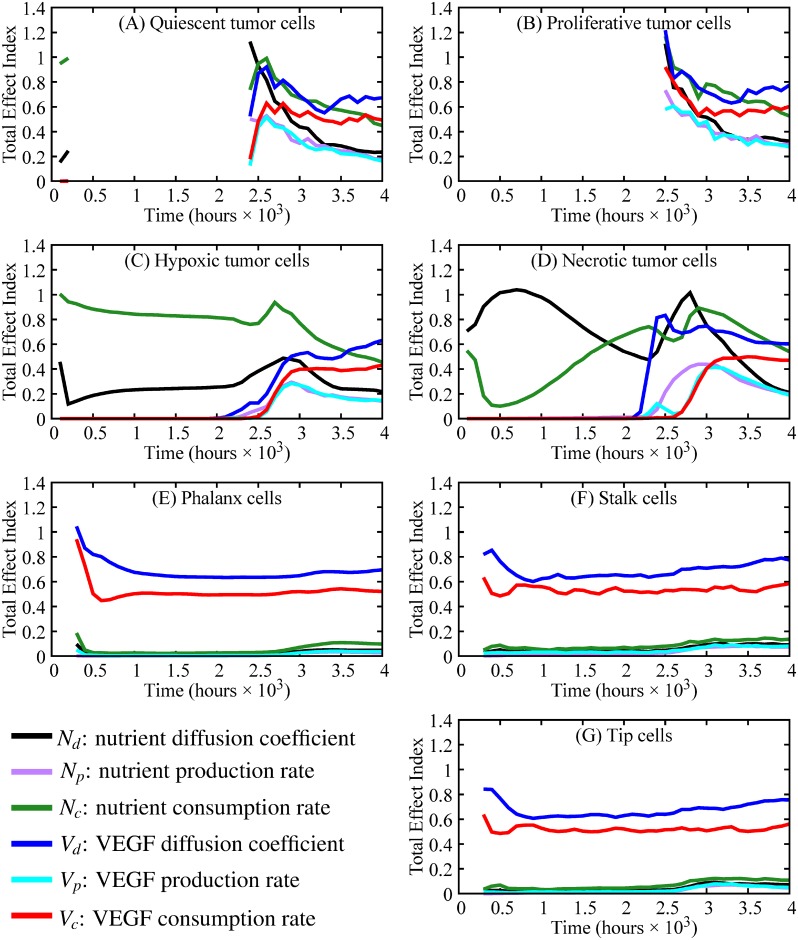
Sensitivity analysis for different tumor cell phenotypes and parameters (black = nutrient diffusion, purple = nutrient production, green = nutrient consumption, blue = VEGF diffusion, cyan = VEGF production, red = VEGF consumption). Panels A-G show the total effect index over time of cell phenotypes with Panels A-D depicting tumor cell phenotypes and Panels E-G depicting endothelial cell phenotypes. The gaps in the total effect index (TEI), most predominantly shown in Panels A and B, occur because TEI is calculated when changes in cell number occur through time (e.g., the number of proliferative tumor cells in Panel B is constant from hour 0 to hour 2900, so there is no TEI). In the beginning stages of tumor growth (hours 0-2500), the nutrient diffusion and nutrient consumption are the most important parameters in tumor composition (i.e., small changes in these parameters would yield large changes in tumor composition). However, after the initiation of angiogenesis and the formation of anastomotic loops, nutrient consumption and VEGF diffusion are the main drivers of tumor composition. Throughout the entire simulations, the most important parameters for angiogenesis and vessel maturation (i.e., vessel growth and anastomosis formation) are VEGF diffusion and consumption.

In Fig 11, the total effect index for each parameter, and for each cell phenotype, is presented every 100 hours from hour 100 to 4000. The number of time steps has been defined to ensure that angiogenesis can occur and that tumor cells are affected by the new source of nutrients. During the initial stages of tumor growth (from 0 to 2400 hours), the most important parameters for the tumor cell phenotypes are nutrient diffusion and consumption (TEI shown in green and black, respectively, in Fig 11, Panels A-D). Once angiogenesis is activated and the new vessels reach the tumor (hour 2400 to hour 4000), the two most influential parameters to tumor cell outcomes are VEGF diffusion and nutrient consumption. In Panels E-G, the parameters with the greatest TEI throughout the entire simulation are VEGF diffusion and consumption. All other parameters have *TEI* < 0.2 throughout the simulations.

## Discussion

In this contribution, we have developed a novel mechanism-based mathematical model to quantitatively characterize angiogenesis by explicitly incorporating vessel occlusion and severing due to the physical interaction between the tumor and endothelial cells. In this new approach, the model computes the physical forces acting on the vessels and the resulting variation in nutrient delivery, growth and composition of the tumor. Furthermore, the model is able to describe the following characteristics of angiogenesis. First, the model recapitulates tip cell activation and angiogenic sprouting due to local tumor hypoxia (Fig 9). Panels B, E, and H depict hypoxia in the tumor region (blue corresponds to a depletion of nutrients) while Panels A, D, and G depict the progression of sprouting in the ABM in response to the VEGF field, shown in Panels C, F, and I. Second, the transition from avascular to vascular tumor growth is captured by characterizing the depletion of nutrients in the tumor microenvironment and the delivery of nutrients by vessels that have anastomosed and the corresponding increase in tumor cell proliferation. The transition of proliferative/quiescent tumor cells to hypoxic tumor cells is shown in Figs 8 and 9, where Fig 8 depicts a proliferative and quiescent tumor (green and blue cells, respectively), while Fig 9 shows a hypoxic and necrotic (orange and gray tumor cells, respectively) tumor. Fig 10 depicts vessel anastomosis and the associated nutrient delivery, and the transition from avascular to vascular tumor growth due to anastomosing vessels. Finally, the model characterizes vessel occlusion/collapse due to the rapid proliferation of newly vascularized tumor regions. Fig 10 depicts the occlusion and collapse of vessels due to the increased forces acting on the vessels from tumor cell proliferation. Panels A, D, and G depict the complex vascular network where vessel sprouts have anastomosed (Panel D) and delivered new nutrients to the tumor (Panel E). This influx of nutrients causes tumor cell proliferation which imparts mechanical forces on the vessels, causing the occlusion and collapse depicted in Panel G. Thus, this approach to modeling angiogenesis is a novel and quantitative characterization of the physical mechanism between angiogenic sprouting and a growing tumor, as well as the resulting effects on tumor growth and composition.

Other efforts have incorporated angiogenesis into mathematical models of tumor growth by treating the tip cell as an agent which guides the vessel based on the gradient of VEGF [31, 84], or by modeling endothelial cells as a continuous field with chemotactic forces based on tumor angiogenic factors guiding the vessels towards the tumor to deliver nutrients [85–87]. Xu et al. applies a phase-field approach to modeling tumor, nutrient, and angiogenic factor dynamics. The model recapitulates not only avascular tumor growth with an inability to induce angiogenesis, leading to a dormant tumor, and vascular tumor growth after induced angiogenesis, but also the angiogenic switch between these two stages. The computational study of DLL4 blockage, coupled mathematically with tip cell activation and inversely with the amount of nutrient delivered by vessel, compares well with experimental studies [31]. Soltani *et al*. utilize a reaction-diffusion model for endothelial cells and incorporate the chemotaxis and haptotaxis forces. Blood flow is modeled using a Poiseuille-like flow and vessels dynamically adapt depending on vessel wall shear stress and intravascular pressure [88]. While these tumor growth models inform nutrient delivery from new vessels formed during tumor angiogenesis, they do not explicitly incorporate the physical interactions between the growing tumor and surrounding vasculature. This physical interaction is a key component in correctly modeling the nutrient delivery as mechanical forces lead to variations in nutrient delivery [13]. Compressing forces induce stresses on the blood and lymphatic vessels, effectively reducing the flow cross-section, increasing the resistance to blood flow, and inhibiting the drainage of interstitial fluid in certain regions of the tumor. This fluid accumulates and increases the interstitial pressure, further decreasing local tumor perfusion through the leaky angiogenic vasculature [13]. While we do not explicitly describe nutrient delivery, the present model could be used to characterize dynamic changes in nutrient delivery as follows. Our model incorporates adhesion forces of the endothelial cells on the blood vessels walls, as well as the repulsion forces of the tumor cells as they increase in number. The repulsive forces of the growing tumor subsequently compresses the vessels. Thus, the degree to which the vessels are compressed determines the vessels’ ability to delivery nutrients to the tumor.

Through a sensitivity analysis, we are able to rank the contribution of the model parameters, particularly the parameters from the reaction-diffusion equations for the nutrient and VEGF, to the number of different phenotypes of tumor and endothelial cells. As we compute the sensitivity analysis every 100 hours, we are able to determine how these contributions change over time. Before angiogenesis is initiated, the number of tumor cells is only affected by the properties directly related to the nutrient (i.e., nutrient diffusion and consumption). Since the phenotypic transitions of tumor cells are dictated by the availability of nutrients at the tumor cell position, the parameters governing the nutrient fields are the main drivers of tumor composition during the avascular stage. After vessels begin to anastomose and supply nutrient to the tumor (2400 to 4000 hours), the contribution of the other parameters increases, with the nutrient consumption and VEGF diffusion becoming the highest contributing parameters to the number of tumor cells with TEI above 0.4 from hours 3000 to 4000. Regarding the number of endothelial cells, the parameters that drive and sustain angiogenesis throughout the entire simulation are VEGF diffusion and consumption. In future experimental studies, it will be critical to accurately measure or calibrate VEGF and nutrient diffusion and consumption rates.

While the model is able to represent the effects of physical interactions on tumor growth, the branching of new vessels, lumen formation, and nutrient delivery, there remain several areas which require future study. For example, the heterogeneity of neither the extracellular matrix (ECM) nor vessel permeability are currently not represented. A more realistic characterization of the ECM can have a significant effect on the geometry and function of the growing vasculature; for example, regions of high ECM density can change the drag force acting on the cells resulting in varying sprout growth rates and densities of vasculature [89]. In the current effort, the homogeneous ECM allows us to directly characterize the effects of the VEGF gradient on the direction of new vessel proliferation and migration. Another limitation of our model is that it is currently implemented only in 2D. In 2D, as vessels sprout, form anastomosis, and establish a complex network, they could completely surround regions of tumor cells and restrict the area in which tumor cells may grow. After receiving a new supply of nutrients, these tumor cells will proliferate and eventually occlude or sever the surrounding vessels. In reality, however, tumor cells may proliferate into the 3^rd^ dimension and have little effect on the encapsulating vessels. Thus, the 2D realization of our proposed model may be over-predicting the frequency of vessel occlusion and collapse. Also, extending it to 3D is not straightforward as the lumen formation is driven by repulsion forces between different sides of the vessel wall, with cells on wall X repelling cells on wall Y. In 3D, defining vessel walls for repulsion is not so clear. One possible way forward is to parametrize vessels by a list of nodes and splines, as described in [90], and evolving the vessel structure based on the local VEGF. However, our immediate application for this model is to calibrate it with time resolved, confocal microscopy measurements of tumor and endothelial cell proliferation and migration obtained in pseudo-2D microfluidic chambers [91] and to make predictions in varying conditions (e.g., anti-angiogenic drugs [92], radiation therapies [93], etc.); thus, we are well positioned for modeling this experimental system.

## Conclusion

We have extended the hybrid multiscale avascular tumor growth model presented in [21] to account for angiogenesis by treating each endothelial cell as an individual agent. We used reaction-diffusion equations to model the nutrient and vascular endothelial growth factor fields which inform the decision-making processes in the ABM that governs the development and migration of new blood vessels. Through numerical experiments, we analyzed the sensitivity of the model parameters and their effects on the number of cells in each tumor and endothelial cell phenotype. In summary, we have contributed a hybrid model rigorously characterizing the physical interaction between a tumor and the surrounding blood vessels, allowing the forces of the growing tumor to influence the nutrient delivery of the vasculature.
